# Supplementary figures and images for: Nodal Expansion, Tumor Infiltration and Exhaustion of Neoepitope-Specific Th Cells After Prophylactic Peptide Vaccination and Anti-CTLA4 Therapy in Mouse Melanoma B16
Source: Int J Mol Sci. 2025 Jul 4;26(13):6453. doi: 10.3390/ijms26136453 (PMC12249801; doi:10.3390/ijms26136453)

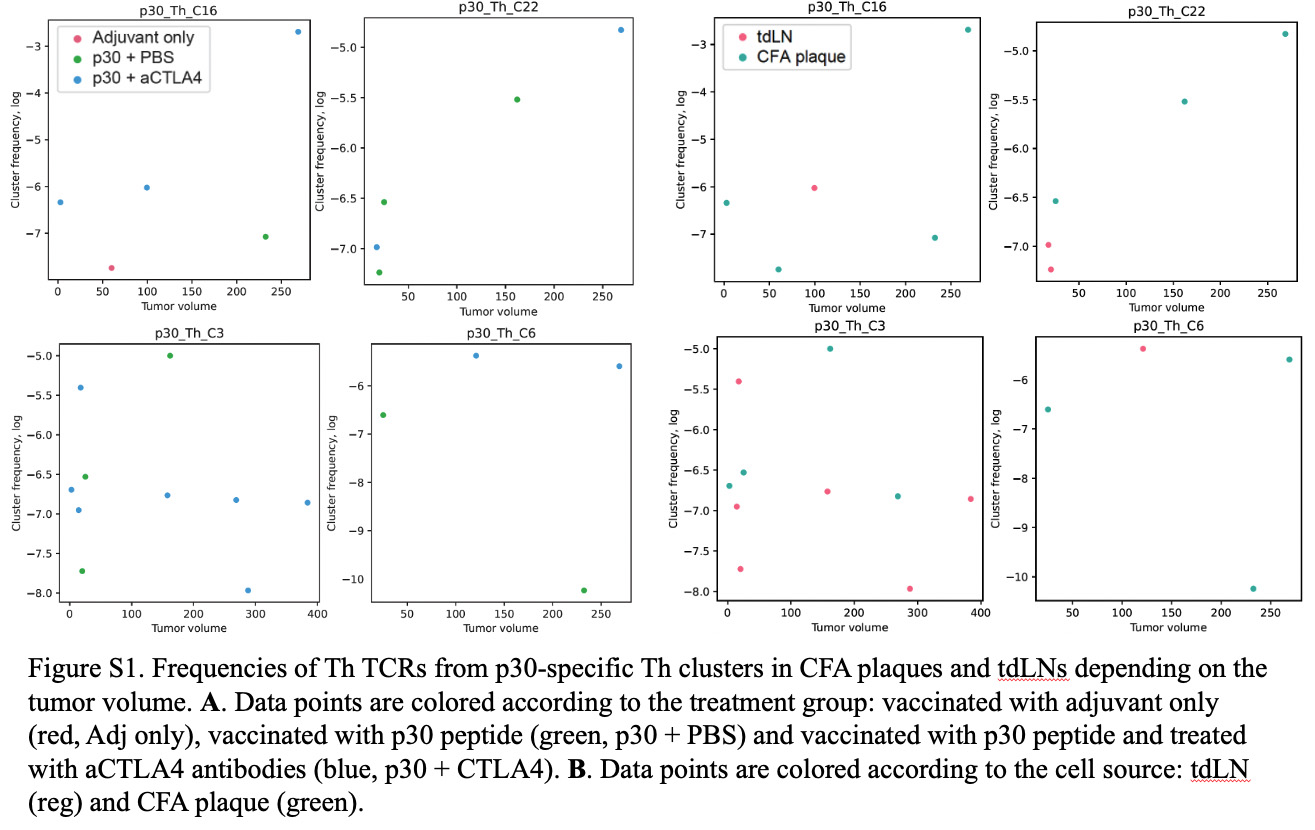

Supplement: Supplementary file 1 [file ijms-26-06453-s001.zip › Shabalkina et.al_FigureS1.jpg]

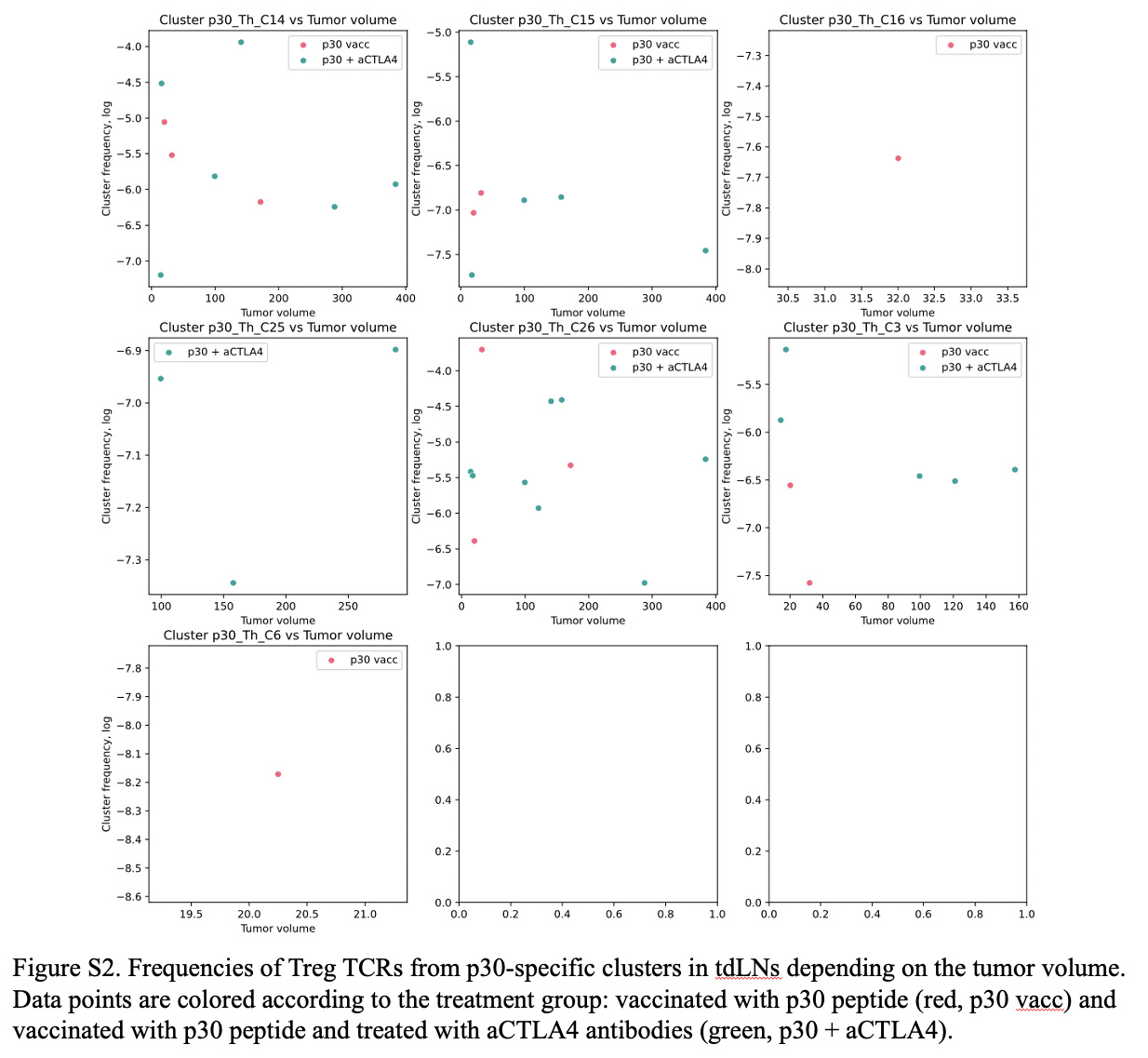

Supplement: Supplementary file 1 [file ijms-26-06453-s001.zip › Shabalkina et.al_FigureS2.jpg]
